# Learning is negatively associated with strength of left/right paw preference in wild grey squirrels (*Sciurus carolinensis*)

**DOI:** 10.3758/s13420-019-00408-2

**Published:** 2020-01-21

**Authors:** Lisa A. Leaver, Steph Ford, Christopher W. Miller, Matilda K. Yeo, Tim W. Fawcett

**Affiliations:** Department of Psychology, Centre for Research in Animal Behaviour, University of Exeter, Exeter, EX4 4QG UK

**Keywords:** Laterality, Lateralization, Paw preference, Learning, Cognition, Comparative cognition

## Abstract

Cerebral laterality, via hemispheric specialisation, has been evidenced across the animal kingdom and linked to cognitive performance in a number of species. Previously it has been suggested that cognitive processing is more efficient in brains with stronger hemispheric differences in processing, which may be the key fitness benefit driving the evolution of laterality. However, evidence supporting a positive association between cognitive performance and lateralization is mixed: data from studies of fish and birds show a positive relationship whereas more limited data from studies of mammals suggest a weak or even negative relationship, suggesting the intriguing possibility of a mammal/non-mammal divide in the nature of this relationship. Here, we report an empirical test examining the relationship between lateralization and cognitive performance in wild grey squirrels (*Sciurus carolinensis*) by measuring left/right paw preference as a behavioural assay of cerebral lateralization and learning speed as an assay of cognitive efficiency. We carried out a motor-based laterality test using a reaching paradigm and measured learning speed on a problem-solving task. In accordance with the suggestion of a mammal/non-mammal divide, we found a negative relationship between strength of paw preference and performance on the learning task. We discuss this finding in light of niche-specific adaptations, task-specific demands and cognitive flexibility.

## Introduction

Lateralization of brain function and structure has, historically, been seen as a mark of advanced cognitive abilities across the animal kingdom, with humans at the pinnacle of the scala naturae (Aristotle, 384–322 BC) both in terms of degree of lateralization and cognitive prowess (Rogers, Vallortigara & Andrew, 2013). This view has been challenged in more recent decades with mounting evidence showing lateralization to be a hallmark of vertebrate and invertebrate brains, and therefore not unique to humans (Frasnelli, Vallortigara & Rogers, 2012; Rogers & Andrew, 2002; Ströckens, Güntürkün & Ocklenburg, 2013). In this way, the history of lateralization research has followed that of animal cognition more generally; nearly a century of research by comparative psychologists and ethologists has led to the generally accepted conclusion that humans do not boast as many unique abilities as previously assumed and the overwhelming majority of scientists in the field now view cognitive abilities as the result of evolutionary processes, leading to similarities across species via shared ancestry or convergent evolution as a result of common selection pressures (e.g. cognitive adaptations for food caching: Smulders, Gould & Leaver, 2010)

However, while it is now known that lateralization is ubiquitous across the animal kingdom, at least in all of the species measured, extreme cerebral lateralization is still taken to indicate cognitive superiority when measured within species; those individuals with more highly lateralized brains typically enjoy a cognitive advantage over those with brains that show a greater degree of bilateral redundancy (Ocklenburg & Gunturkun, 2012; Rogers, 2017). Split-brain studies in humans have shown that more general perceptual processes are bilateral, whereas higher-level cognitive processes are strongly lateralized (Gazzaniga, 2000), which may be due to the dual advantages of enhanced computational speed associated with unilateral processing (Ringo, Doty, Demeter & Simard, 1994) combined with the fact that bilateral processing may result in “intrahemispheric conflict” (Corballis, 2009).

Empirical evidence supports the theory that cerebral lateralization is positively linked to cognitive performance across a broad range of species (Rogers, 2017). In vertebrates, the degree of cerebral lateralization can be measured non-invasively via limb (e.g. paw, hand) or visual preferences due to the contralateral control of action and perception (Vallortigara & Rogers, 2005). Studies directly examining the relationship between behavioural laterality and cognitive performance (usually in specific learning tasks) have been primarily conducted on birds and fish. For example, more highly visually lateralized pigeons, *Columba livia* (Gunturkun, Diekamp, Manns, Nottelmann, Prior, Schwarz & Skiba. 2000), and more lateralized individuals (calculated as a composite of limb and eye preferences) in eight species of Australian parrots (Magat & Brown, 2009) showed improved visual discrimination in a foraging task. For the parrots in the same study, laterality strength was also positively related to performance on a string-pulling task (Magat & Brown, 2009). Similarly, guppies, *Poecilia reticulata,* with stronger side preferences had better numeric discrimination abilities than guppies with less pronounced preferences (Dadda, Agrillo, Bisazza & Brown, 2015). Rogers (2017) provides a comprehensive review of this body of work, concluding that the general relationship is positive.

In addition to a general cognitive advantage, lateralization of function has also been linked to a specific advantage in tasks that require split attention by engaging each hemisphere simultaneously in its own specialist task. Visually lateralized domestic chicks (*Gallus gallus domesticus*; Rogers, Zucca & Vallortigara, 2004), fish (*Cymatogaster aggregate*) with a stronger turning preference (Dadda, Koolhaas & Domenici, 2010) and marmosets (*Callithrix jacchus*) with a stronger left/right paw preference (Piddington & Rogers, 2013) performed better than less lateralized individuals when required simultaneously to discriminate food and detect predators. Gazzaniga (2000) found that split-brain humans were better than intact controls at doing tasks that required divided attention, but worse where the tasks required hemispheric collaboration.

Underlying the purported improved performance of more strongly lateralized individuals, it has been proposed that cognitive processing is more efficient in brains with stronger hemispheric differences in processing, and this efficiency may be the key fitness benefit driving the evolution of laterality (Rogers et al., 2013). However, the focus on the positive relationship between laterality and cognition has somewhat overshadowed at least two lines of evidence indicating that there are costs associated with lateralization. The first is the persistence of individual variation in the strength of lateralization – to the best of our knowledge, there are no species where lateralization has driven to fixation (reviewed by Ghirlanda & Vallortigara, 2004). The second line of evidence consists of a handful of studies showing that strength of lateralization is related to negative fitness outcomes associated with lower life expectancy (Whiteside, Bess, Frasnelli, Beardsworth, Langley, van Horik & Madden, 2018), suboptimal decision making (Dadda, Zandona, Agrillo & Bisazza, 2009) and reduced foraging efficiency (Miler, Kuszewska, Zuber & Woyciechowski, 2018). The costs associated with lateralization are further indicated by the finding that lateralization strength in poeciliids decreases as predation pressure is relaxed (Brown & Braithwaite, 2005; Brown, Gardner & Braithwaite, 2004). The mix of costs and benefits associated with laterality is indicative of a trade-off faced by animals in their natural environments (Frasnelli & Vallortigara, 2018).

Perhaps surprisingly, we have found only three published studies directly measuring learning performance in relation to behavioural laterality in non-human mammals. The first is a study by Hörster and Ettlinger (1985) in rhesus monkeys (*Macaca mulatta*), which showed the opposite result to studies on fish and birds: ambidextrous monkeys were significantly faster at learning a tactile discrimination task than those with a left- or right-hand preference. The second study, looking at left/right paw preference in dogs (*Canis familiaris*), also reported a learning speed advantage for non-lateralized individuals (Marshall-Pescini, Barnard, Branson & Valsecchi, 2013). The third study, on marmosets, reported no relationship between left/right paw preference and learning speed on a foraging task (Piddington & Rogers, 2013). In humans, the only other mammal that seems to have been studied, results are mixed and hotly debated (see Hirnstein, Leask, Rose & Hausmann, 2010, for a good summary of the debate). A meta-analysis by Nettle (2003) indicates that while there is a significant increase in general cognitive ability with increased laterality scores, this effect – which accounts for less than 1% of the variance in IQ – is so weak as to be of negligible importance. Taken together, these studies suggest the intriguing possibility that mammals may differ from the rest of the animal kingdom in showing no clear relationship or even a negative relationship between laterality and cognitive performance, rather than a positive one. However, the data are sparse, and more study is required.

The aim of our study was to examine for the first time the relationship between laterality and cognitive performance in a rodent, the Eastern grey squirrel (*Sciurus carolinensis*), to determine whether the direction of this relationship further supports the possibility of a mammal/non-mammal divide. All of the studies reporting links between behavioural lateralization and cognition have been conducted on captive animals tested in laboratory conditions, while there is a notable lack of data from wild animals living under natural conditions. To understand the conflicting adaptive pressures that may have led to the evolution of lateralization, it is important to take more direct measures of the real-life costs and benefits of lateralization faced by animals. Our second aim, therefore, was to conduct the first test of lateralization and learning performance in the wild. Eastern grey squirrels are an ideal species for testing such a relationship not only because there are no such studies in the Rodentia, but also because they are excellent problem solvers both in terms of their anecdotal prowess in accessing garden feeders and their excellence in problem solving under experimental conditions (e.g. Chow, Lea & Leaver, 2016).

We were particularly interested to see whether the relationship between behavioural lateralization and cognitive performance in wild grey squirrels fell in line with previous findings in fish and birds, where individuals with stronger behavioural lateralization have a cognitive advantage (as measured by performance on a learning task), or instead fitted with the handful of results from other mammals, where the cognitive advantage lies, if anything, with less lateralized individuals. To test this relationship, we measured the behavioural bias of wild squirrels on a motor-based laterality test, using a reaching paradigm as an assay of cerebral lateralization, and their learning speed on a problem-solving task as an assay of cognitive efficiency.

## Methods

The study was carried out between January 2017 and July 2019 on the University of Exeter’s Streatham Campus (50. 721800, -3.533620; 50^0^ 43' 18.48” N, 3^0^ 32' 1.032” W) using wild Eastern grey squirrels. Two wooded parkland sites, each approximately 2 hectares in size, were used: Reed Hall and Birks Bank. Squirrels were captured, given unique markings using black hair dye (Clairol^TM^) and released at the site of capture under Natural England Non-Native Species Release License 2018-36426-SPM-NNR (for details of the habitat, trapping and marking process, see Leaver, Hopewell, Caldwell & Mallarky, 2007). A total of 31 squirrels (18 male, 13 female) were marked over the course of this study.

Within each of the two sites, ten sheltered locations were regularly baited with a small handful of raw shelled peanuts (~85 g) at least 5 days per week throughout the study to encourage the squirrels to habitually visit fixed sites. The study apparatus was set up at the Reed Hall site at two locations in both years (though the location of one site was moved in 2019), two locations at Birks Bank in 2018 and one location at Birks Bank in 2019. Apparatus was placed out 3–5 days per week from January 3 until July 30 each year.

**Fig. 1:**
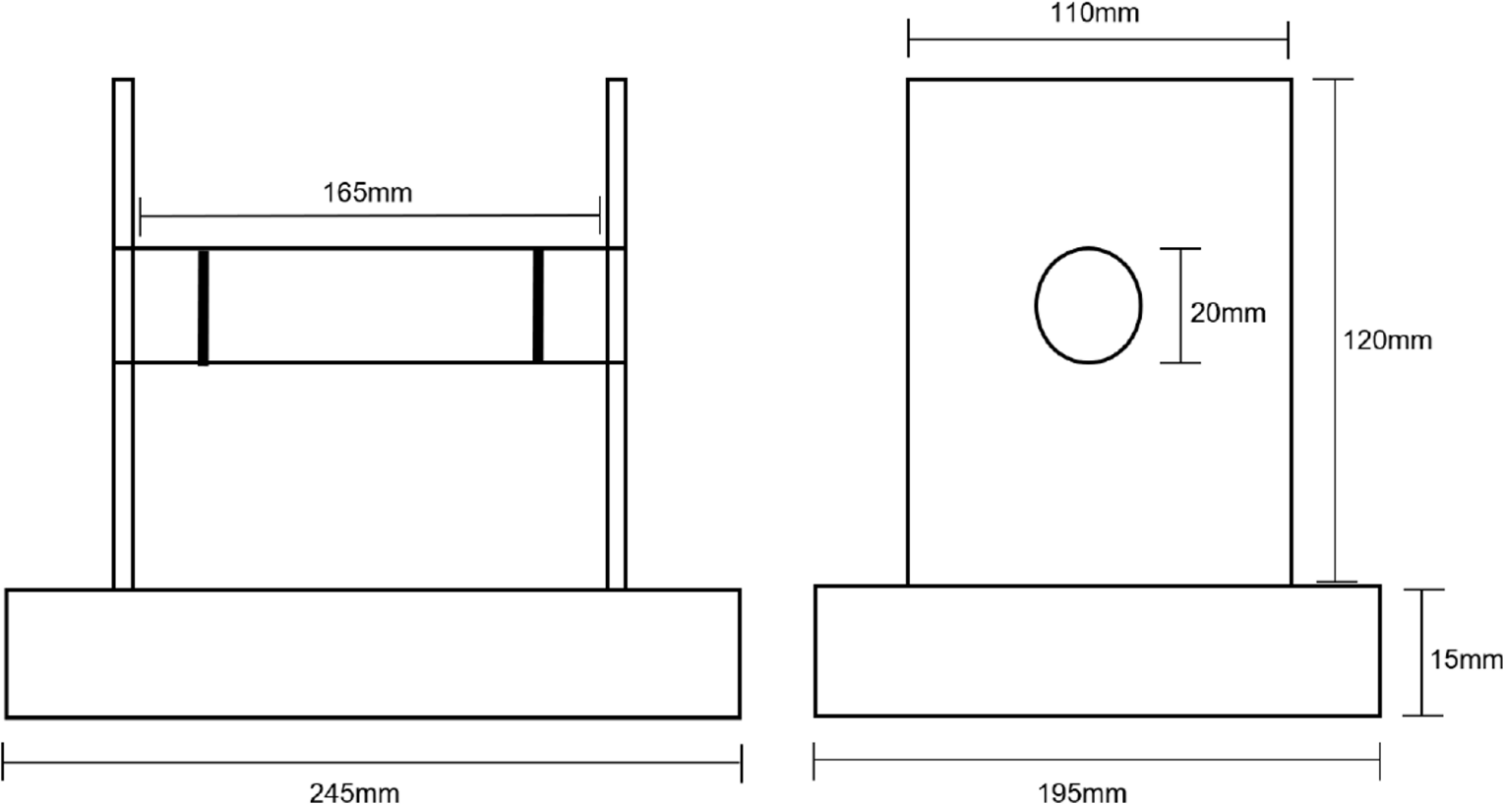
Schematic representation of the tube apparatus showing relevant dimensions

The apparatus consisted of a horizontal clear Perspex tube supported on a wooden base pegged to the ground to prevent the squirrels from lifting it up to tip out the nuts (Fig. 1). Video recorders (Panasonic HC-W580) were set up on tripods to record all activity at the apparatus for later analysis. Daily presentation of the apparatus was maximised depending on the battery life of each camera, which ranged from 2 to 5 h. Squirrels were allowed to become accustomed to the apparatus over the course of approximately 3 weeks with peanuts scattered around it and across the base, but not in the Perspex tube. Because squirrels visited at different intervals, it was not possible to standardise this for all participants, but all visiting squirrels were quick to associate the apparatus with food, and all visiting squirrels overcame initial neophobia and participated in at least some trials.

**Fig. 2:**
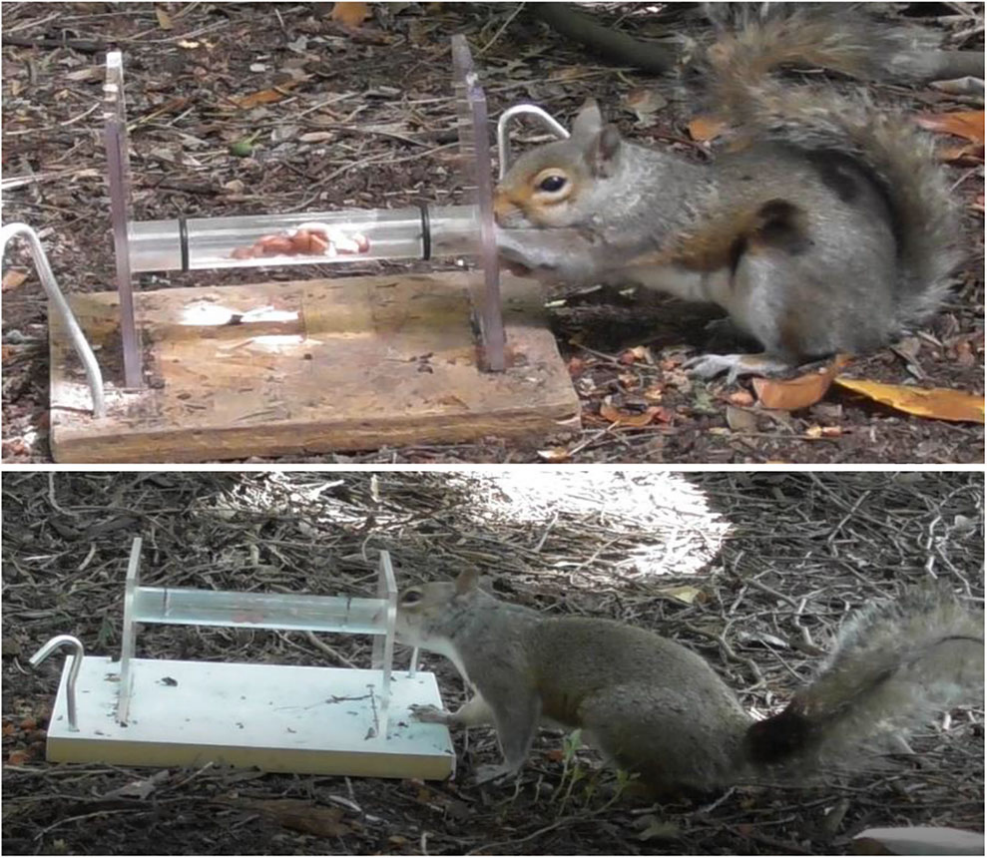
**Top:** BF2E uses a paw to reach into the tube. **Bottom:** BABC performs an unsuccessful head attempt

Once squirrels were reliably collecting peanuts from the base of the apparatus (ranging from one to three visits per squirrel), the experimental trials began. The cylinder entrance was 20 mm in diameter, too narrow to allow a squirrel to insert its entire mouth, and the peanuts were placed 25 mm into the tube to rest between the black lines, beyond the squirrel’s limited mouth reach. Peanuts in this position were only accessible to squirrels via reaching with their paws (Fig. 2). Squirrels have a strong tendency to use their mouths rather than their paws to obtain food, and all squirrels’ initial attempts to obtain nuts were with their mouth. In their first ten attempts, paw use scores for all squirrels ranged from zero to 2 out of 10.

All video data were scored separately by different authors for handedness and learning using the observational coding software BORIS (Friard & Gamba, 2016). Handedness was scored for each participating squirrel by recording which paw was used to reach into the tube, regardless of whether the attempt was successful in obtaining a nut. A minimum of 50 up to a maximum of 1,640 reaches were scored per squirrel and individual laterality index (LI) scores were calculated using the following equation: 100 × (*L*−*R*)/(*L*+*R*), where *L* and *R* are the number of left-pawed and right-pawed attempts, respectively (adapted from Batt et al., 2008; Wells, 2003). This gave each squirrel a score between −100 and +100, with scores of 0 indicating ambidexterity, increasingly positive scores indicating more extreme left-paw bias and increasingly negative scores indicating more extreme right-paw bias. We calculated the strength of lateralization (ignorant of direction) by taking the absolute value of each LI score (ABS-LI), thus rendering all scores between 0 and 100 for analysis (Rogers, 2017).

Learning scores were calculated by scoring each attempt to obtain a nut from the tube as either head or paw. An ‘attempt’ was defined when any part of a squirrel’s muzzle or paw was inserted into the mouth of the tube beyond the rim and was terminated when the muzzle or paw was withdrawn. One entrance in and one exit out of the tube by the mouth or paw constituted a single attempt regardless of the extent of movement made inside the tube. A ‘mouth attempt’ was coded when any part of a squirrel’s muzzle or tongue was inserted into the tube, including any movement of the mouth thereafter including chewing, licking, sniffing or biting. A mouth attempt was not counted if the mouth was inserted after a paw to receive a nut successfully retrieved by a paw. A ‘paw attempt’ was coded when a paw was inserted beyond the rim of the tube, and included any pushing, pulling or nudging of a nut towards the mouth. Each attempt was marked as either ‘success’ or ‘fail’ depending on whether the squirrel obtained a peanut or not. For each squirrel, attempts were grouped in blocks of ten and the total number of paw attempts out of ten was recorded to quantify learning over trial blocks. The learning criterion was set as three successive blocks of eight to ten paw attempts.

We computed a learning curve for each squirrel using proportional logistic regression, in which the proportion of paw attempts (out of ten) was modelled as a function of the trial block. We then used the fitted model to predict performance (i.e. proportion of paw attempts) in block 25, one more trial than the maximum allowed for learning to criterion. This method allowed us to include in the analysis those squirrels that did not learn the task to criterion, therefore demonstrating poor cognitive performance (see Langley, van Horik, Whiteside & Madden, 2018, for a similar approach). We analysed the correlation between this predicted performance measure and the ABS-LI score to assess the relationship between cognitive performance and lateralization.

All statistical analyses were run using IBM SPSS v25 (IBM Corp., 2017) and R/RStudio (R Core Team, 2018).

This research was reviewed and approved by the Psychology Ethics Committee (ethics application eCLESPsy000176v4.0).

## Results

A total of 12 squirrels participated in this experiment: nine marked squirrels (four male, five female) plus three others that were individually identifiable (one female had half a tail, one male had a ‘chunk’ out of one side of its tail, and one squirrel of unknown sex had a distinctively orange tail). After 28 trial blocks, the orange-tailed squirrel had not only failed to reach criterion, it had also only used its paw once, so we could not generate a laterality index for this squirrel and it was excluded from the analysis.

**Fig. 3:**
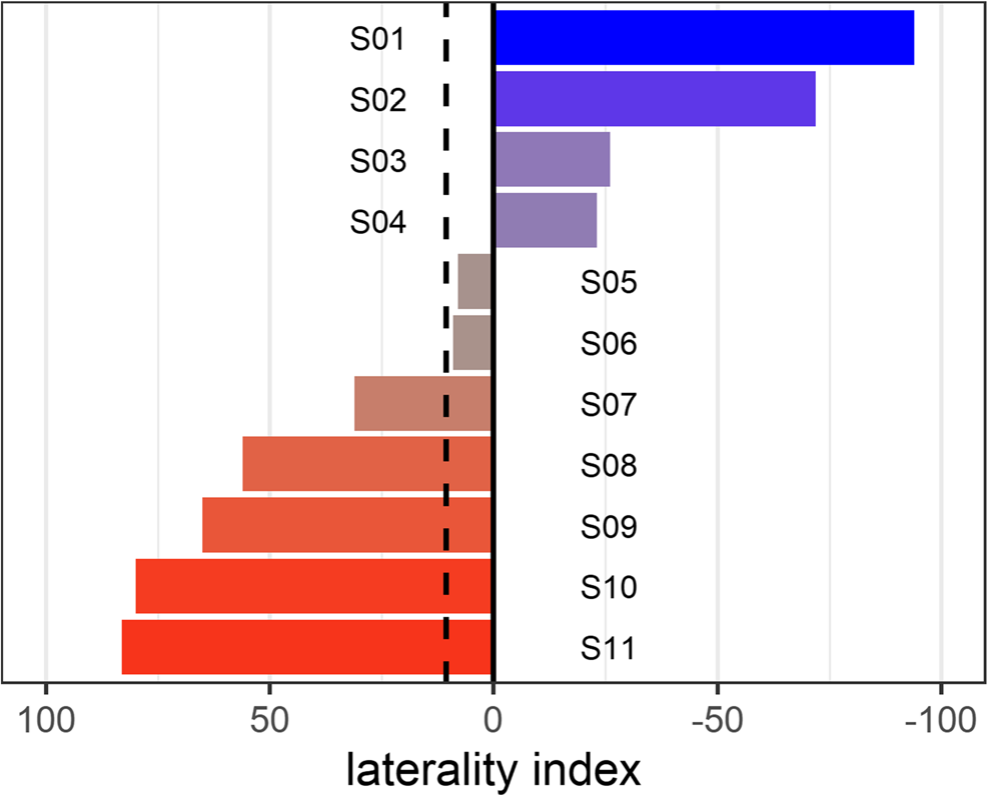
Laterality index scores for each squirrel. Dashed line shows mean value. Increased colour saturation indicates stronger lateralization, with blue indicating right-paw bias, red left-paw bias and grey ambidexterity

Laterality indices for the remaining 11 squirrels ranged from −94 to 83 with a mean of 10.64 and a median of 9, indicating a slight but non-significant tendency towards left paw usage at the group level (one-sample *t*-test: *t*_10_ = 0.590, *p* = 0.568; Fig. 3).

**Fig. 4:**
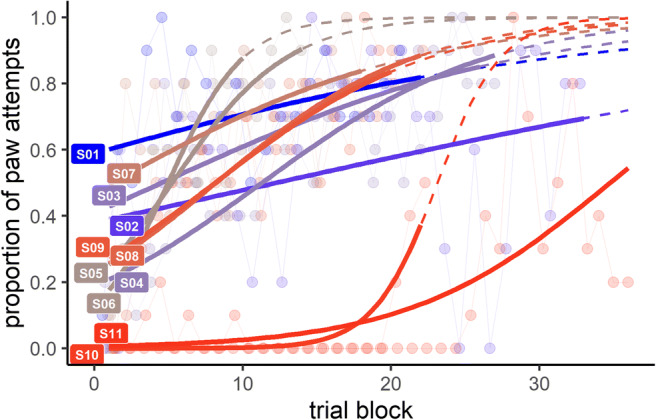
Individual learning curves for each squirrel. Solid lines in the foreground show the fitted curve for each squirrel from a logistic regression modelling the proportion of paw attempts in each bout of ten trials, with the raw data shown as dots and connecting lines in the background. Dashed lines indicate predicted performance beyond the observed trials (based on extrapolation of the logistic curve). Colours for each squirrel correspond to those in Fig. 3

Nine of 11 squirrels (82%) reached the learning criterion between block 10 and block 24 (Fig. 4).

**Fig. 5:**
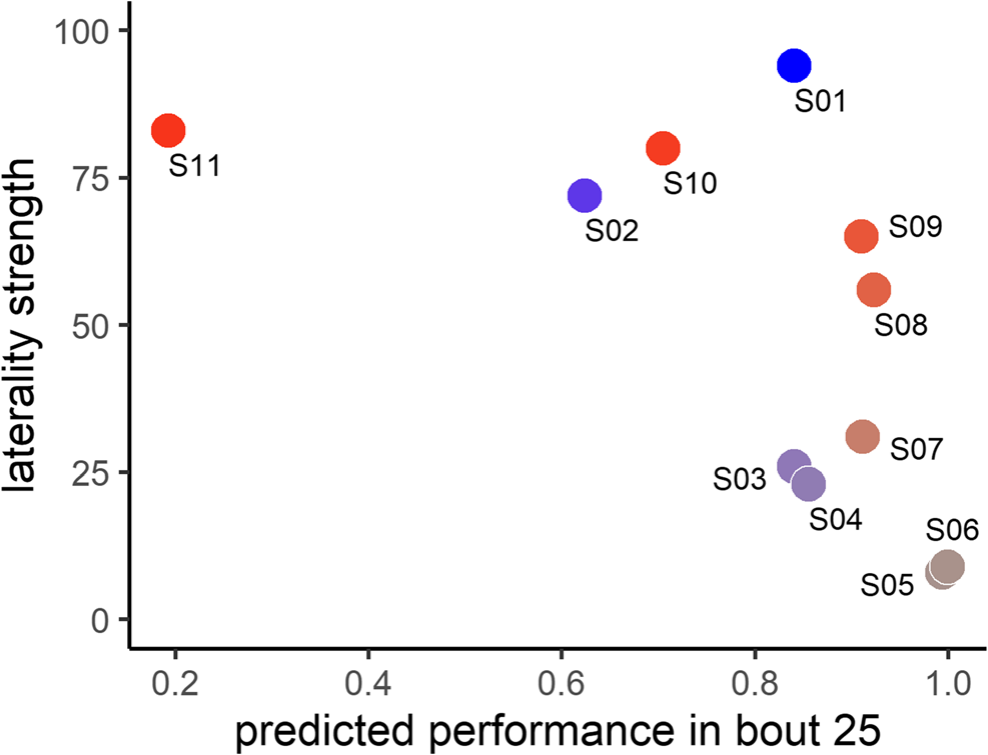
Relationship between strength of laterality (AMB-LI) and predicted performance in bout 25 (as predicted from the learning curves in Fig. 4). Colours for each squirrel correspond to those in Fig. 3

The number of trials to criterion is a commonly used measure of learning performance, but is problematic here because there were two squirrels that failed to reach criterion. Excluding these individuals would eliminate important data, as the non-learners are at the extreme of poor cognitive performance and thus particularly interesting for our research question. One possible solution would be to allocate an arbitrarily high score to non-learners, but this is potentially misleading because the choice of value influences the relationship of interest. Furthermore, as can be seen from the performance of S02 in Fig. 4, trials to criterion does not perfectly capture learning performance because despite reaching criterion at block 17, his performance dropped below criterion for all subsequent trials (terminated at block 33). To overcome these issues, we compared the squirrels’ performance at an equivalent point in time, specifically block 25, predicted on the basis of a proportional logistic regression fitted to their learning data (curves shown in Fig. 4). This revealed a significant negative association between the strength of laterality (ABS-LI score) and predicted performance at block 25 (Spearman correlation: *r*_*S*_ = −0.718, *n* = 11, *p* = 0.013; Fig. 5).

## Discussion

Our results show that strength of paw preference in a reaching task was negatively related to learning speed in squirrels, i.e. squirrels with stronger side biases showed a slower improvement in task performance across trials. Our study provides the first test of this relationship in a rodent, as well as the first test in the wild. Our findings fit with the negative relationships found in other non-human mammals, specifically rhesus macaques and dogs, but run counter to the positive relationship found in birds and fish, consistent with our suggestion of a mammal/non-mammal divide. The possibility that mammals do not gain a similar cognitive advantage from strong cerebral lateralization to birds and fish suggests that the costs and benefits associated with strong lateralization of brain function and more symmetrical bilateral control are finely balanced and closely linked to ecological challenges. While the suggestion of a divide across the animal kingdom is intriguing, there is no *a priori* reason to expect other advantages to hemispheric laterality across broad taxonomic groups and, in fact, adaptive specialisations such as those linked to tool use in primates (Sfar, Mangalam, Kaumanns & Singh, 2014) are more likely to have arisen under a history of niche-specific selection pressure.

Stronger lateralization, through hemispheric specialisation and modularity of function, offers high-speed processing and expansion of cognitive function by avoiding bilateral redundancy, whereas a less lateralized brain may offer the safer, risk-averse advantages of redundancy through bilateral processing (Ringo et al., 1994). Grey squirrels, by dint of being food cachers, occupy an ecological niche that has been well studied in terms of its related cognitive and behavioural adaptations. Grey squirrels are heavily reliant on spatial memory to recover thousands of food caches over the course of weeks or even months (Macdonald, 1997) and, as such, may benefit from the redundancy of bilateral processing provided by a more symmetrical brain. Short-term memory, particularly free recall, requires bilateral processing or at least intrahemispheric communication (Phelps, Hirst & Gazzaniga, 1991), which is of particular importance for species like grey squirrels whose survival depends on recollection of spatial locations. Brown and Braithwaite (2005) showed that fish with stronger turning biases suffered in their ability to navigate through a maze, and Tommasi and Vallortigara (2001) showed that, in chicks, both hemispheres were required to encode global and local cues during navigation, providing further tentative evidence that spatial navigation may be linked to hemispheric laterality. While it is tempting to suggest that the cognitive requirements of food caching provide a functional explanation for our results, there is also evidence showing hemispheric specialization of spatial memory in birds (Clayton & Krebs, 1994), so the answer is probably less clear-cut.

The cost-benefit balance associated with the degree of hemispheric lateralization likely differs not only between species, due to niche-specific adaptations, but also within species, depending on task-specific demands (Rogers, 2009). Task-specific advantages of lateralization have been shown in fish (*Girardinus falcatus*) choosing a shoal (Dadda et al., 2009), chicks attending simultaneously to food and predators (Rogers et al., 2004), chimpanzees (*Pan troglodytes schweinfurthii*) fishing for termites (McGrew & Marchant, 1999), desert locusts (*Schistocerca gregaria*) stepping over a gap (Bell & Niven, 2016) and across a variety of primate species during feeding, tool use and communication (reviewed by Versace & Vallortigara 2015), leading to the perhaps unsurprising conclusion that different tasks demand different degrees of hemispheric specialization. Our results are reliant on just one test of cognitive performance, which is fairly typical of the non-human animal work cited here (but see Whiteside, Bess, Frasnelli, Beardsworth, Langley, van Horik & Madden, this issue). To directly address the question of task-specific performance in relation to lateralization strength it is imperative for more researchers to conduct a range of tests across individuals of the same species.

Is cognitive flexibility the key to this apparent trade-off? Perhaps the efficiency of processing associated with stronger hemispheric specialisation is offset by lack of flexibility in those responses. As grey squirrels survive on seasonally available food and rely on effective scatter-hoarding, behavioural flexibility may be important both to facilitate switching between fluctuating food sources and to inhibit immediate consumption of those foods needed for future use. Furthermore, successful invasive species thrive in part because of their flexible behavioural innovations (Sol, Timmermans & Lefebvre, 2002). The grey squirrel’s ability to survive and reproduce in novel environments since its introduction to the United Kingdom may be due to behavioural flexibility across cognitive and foraging contexts, although flexibility has also been found to vary among individuals in this species (Chow, Lea & Leaver, 2016). If individual cognitive flexibility is the key to learning (Lea, Chow, Leaver & McLaren, this issue), then perhaps the individual differences in learning that we report here are related to individual differences in cognitive flexibility. This would not be the first suggestion that symmetrical bilateral processing may be associated with more flexible thought and behaviour; Corballis (2009; p. 875) commented, in relation to humans, that:

“A symmetrical brain may well provide avenues of thought that do not conform to academic expectations, but may nonetheless provide the impetus for significant discovery and leadership…We have seen that those without consistent handedness, for example, may differ from both the left- and right-handers in terms of both intellectual abilities and personality characteristics such as magical ideation, delusional behaviour and possibly creativity. This is one avenue, I suggest, that it might be useful to explore further.”

Our findings address the gap in the literature that Corballis identifies, by providing further evidence from a non-human animal suggesting that, in mammals at least, there may be advantages in terms of more flexible cognitive processing associated with reduced cerebral asymmetry (see also Found & St. Clair, 2017).

While it is interesting to speculate about the broad implications of our findings, there are of course some limitations to our work, and further study is required to confirm or refute any of our suggestions. Testing squirrels in the wild is labour intensive and requires marked individuals to visit baited sites on a regular basis. Only nine of 31 marked individuals returned to participate in this study, and it is likely that they represent a self-selected subset in terms of boldness, aggression or some other characteristic that may also set them apart in terms of cognitive ability (van Horik, Langley, Whiteside & Madden, 2017) and lateralization (e.g. Reddon & Hurd, 2009). While testing in the wild increases ecological relevance, our tube task itself is of questionable validity since squirrels rarely, if ever, use their paws to obtain food. Squirrels preferentially use their mouths to manipulate objects in a variety of contexts from foraging to masturbation (Waterman, 2010), so further work measuring learning and performance in more natural tasks is needed to determine whether lateralization strength in squirrels is important more generally for learning. Furthermore, as well as determining whether the nature of this apparent trade-off persists across tasks, future studies should ideally measure whether and how laterality strength relates to outcomes that are relevant to fitness such as foraging efficiency, reproductive success and life expectancy.

The aim of this study was to gain insight into the adaptive value of lateralization by studying its links to cognition via learning in a small mammal in a wild setting. Our findings show that in grey squirrels, like other non-human mammals studied, strength of paw preference in a task involving paw use is negatively related to cognitive performance on a learning task, calling into question the common claim from research on fish and birds of a cognitive advantage associated with increased strength of laterality. Our findings call for further research into the cognitive and fitness trade-offs associated with varying degrees of laterality across a broad range of species, to gain a more complete understanding of how selection acts on lateralization.

### Open Practices Statement

The dataset generated and analysed during the current study is available from the first author upon reasonable request. This study was not preregistered.
